# Analytical validation, reference interval determination, and diagnostic performance of plasma symmetric dimethylarginine in cattle

**DOI:** 10.1093/jvimsj/aalag108

**Published:** 2026-06-09

**Authors:** Charly De Campos, Inês de Brito Neves Barroca, Zahida Baydoun, Théo Chenal, Manon Gonçalves-Brandão, François Schelcher, Catherine Trumel

**Affiliations:** Département Elevage et Produit, Santé Publique Vétérinaire, Ecole Nationale Vétérinaire de Toulouse, Université de Toulouse, Toulouse, France; CREFRE, Université de Toulouse, INSERM, UPS, ENVT, Toulouse, France; CREFRE, Université de Toulouse, INSERM, UPS, ENVT, Toulouse, France; CREFRE, Université de Toulouse, INSERM, UPS, ENVT, Toulouse, France; Département Elevage et Produit, Santé Publique Vétérinaire, Ecole Nationale Vétérinaire de Toulouse, Université de Toulouse, Toulouse, France; IHAP, Université de Toulouse, Toulouse, France; CREFRE, Université de Toulouse, INSERM, UPS, ENVT, Toulouse, France

**Keywords:** cattle, diagnostic performance, kidney disease, reference interval, SDMA, validation

## Abstract

**Background:**

The efficacy of serum urea and creatinine concentrations in the diagnosis of kidney disease in cattle is limited because of their low sensitivity and specificity. Serum symmetric dimethylarginine (SDMA) concentration is correlated with glomerular filtration rate and has been reported to be useful for the diagnosis of kidney disease in other species.

**Hypothesis/Objectives:**

Test the hypothesis that SDMA may be an effective biomarker for diagnosing kidney disease in cattle. The objectives were to assess the analytical performance of SDMA measurement using a benchtop analyzer to establish an SDMA reference interval in cattle, and to evaluate the efficacy of plasma SDMA concentration for the diagnosis of kidney disease in cattle.

**Animals:**

A total of 199 healthy animals (53 male and 96 female beef cattle and 50 female dairy cows) and 27 azotemic cows with confirmed kidney disease.

**Methods:**

A partial analytical validation of SDMA measurement using bovine plasma was performed including assessments of short- and long-term imprecision, linearity, and stability. The reference interval was established following American Society for Veterinary Clinical Pathology (ASVCP) recommendations. Finally, the diagnostic efficacy of SDMA was assessed by determination of specificity, sensitivity, and the area under the receiver operating characteristic curve.

**Results:**

The SDMA measurements showed acceptable analytical performance. The reference interval was 60-160 μg/L. No significant differences were found according to sex, breed, or food supply. Using the upper limit of the reference interval (160 μg/L) as the cut-off, sensitivity and specificity were 0.85 and 0.98, respectively.

**Conclusions and clinical importance:**

An SDMA cut-off of 160 μg/L shows promise for diagnosing kidney disease in cattle, but requires confirmation in future studies.

## Introduction

Data from slaughterhouses indicate that the prevalence of kidney disease in cattle can range from 4% to 10%.^1^^,^^2^ Although the overall prevalence of kidney disease in cattle is relatively low, these conditions remain clinically important to recognize and treat, especially in older animals. The most commonly observed renal lesions include pyelonephritis, interstitial nephritis, glomerulonephritis, and acute tubular necrosis.^1^^,^^2^ In practice, diagnosis is primarily based on urinalysis^3^ and measurement of serum or plasma creatinine and urea concentrations.^4^ Additional diagnostic procedures, such as renal ultrasonography or evaluation of the urine protein-to-creatinine ratio (UPC), also may be performed.^5^

However, serum or plasma creatinine and urea concentrations in cattle are influenced by multiple factors, lowering their diagnostic effectiveness. Serum or plasma creatinine concentration is affected by muscle mass, which varies by breed and sex,^6^^,^^7^ whereas urea concentration depends on dietary factors, especially crude protein intake.^8^ Moreover, in cats and dogs, these biomarkers have a low sensitivity, because at least 75% of nephrons must be nonfunctional before serum creatinine concentration increases above the upper limit of the reference interval.^4^

Symmetric dimethylarginine (SDMA) is an endogenous methylated derivative of the amino acid arginine, eliminated almost exclusively by glomerular filtration without tubular reabsorption. Since the early 2010s, SDMA has gained increasing attention in veterinary medicine as a potential biomarker of renal function across species.^4^ In dogs and cats, and in a limited number of studies in horses,^9–11^ SDMA has been reported to correlate with glomerular filtration rate (GFR), supporting its use as a marker of renal function. Although SDMA does not consistently increase earlier than serum creatinine concentration, its independence from muscle mass^10^^,^^11^ offers a clinical advantage, particularly in geriatric or cachectic animals, or in those with high muscle mass (eg, athletic horses, working or sporting dogs) as well as in those with muscle wasting. In contrast, studies investigating the relevance of SDMA in small ruminants have yielded inconsistent results.^12^^,^^13^

Our objectives were to ensure that the analytical characteristics of the SDMA method of a widely used veterinary benchtop analyzer were satisfactory for bovine plasma; to establish a reference interval for plasma SDMA concentration in cattle while accounting for the possible effects of sex, type of production, and diet; and finally, to investigate its diagnostic efficacy for the diagnosis of kidney diseases in cattle.

## Materials and methods

The protocol of our prospective study was approved by the “Science et Santé Animale” ethics committee (SSA-2025-011_V3). All breeders signed a consent form.

### Description and selection of the reference sample group

To be as representative as possible of different French husbandry systems, cattle were selected from 119 herds located throughout western, central, and southwestern France. Cows were from 72 herds, whereas bulls represented 47 herds. High-yielding dairy cows were fed predominantly (95%-100%) on corn silage (CS) or grass silage (GS) with occasional supplementation (<5%) with wheat- or barley-based concentrates. Beef cows and bulls were fed exclusively grass (G) and hay (H). Water was provided ad libitum to all animals. All herds were free from bovine leukemia virus, brucellosis, tuberculosis, bovine viral diarrhea, and infectious bovine rhinotracheitis. Bulls were chosen at a national cattle competition in southwest France (Les Pyrénéennes, Villeneuve de Rivière, France), and represented 6 beef breeds and 2 dairy breeds. To avoid a herd effect, a maximum of 2 bulls and 5 cows at one of 3 stages of lactation (ie, early lactation [0-90 days in milk], mid lactation [90-180 days in milk], or dry period) were included from each herd. Sampling was performed from September to December 2024.

The health status of each animal was evaluated based on a complete clinical examination, including udder and reproductive assessments, and a comprehensive questionnaire addressing current health, dietary intake, medical history over the preceding 2 months and, for dairy cows, both individual- and herd-level production. To assess kidney function, plasma concentrations of urea, creatinine, and SDMA were measured, and complete urinalysis (dipstick analysis, urine specific gravity [USG], urinary cytology, and UPC measurement) was performed. For the purpose of evaluating health condition, the following variables were measured in plasma: total protein, albumin, total calcium, inorganic phosphate, magnesium, creatine kinase, aspartate aminotransferase (AST), and gamma-glutamyl transferase (GGT), and a CBC was performed. Exclusion criteria were medical treatment or any clinical disease during the previous 6 weeks, or any abnormality identified during the complete physical examination performed before sampling, which included rectal temperature, respiratory, cardiac, and ruminal assessments, and routine blood testing and urinalysis.

All cattle included were at least 2 years old to prevent an age effect, which has been shown in dogs, cats, and horses for SDMA.^6^^,^^7^

All analyses except for UPC and the biochemistry panel were performed by a mobile clinical pathology laboratory, previously validated for specimens from dogs, and equipped with Catalyst One and ProCyte Dx analyzers (IDEXX Laboratories, Westbrook, ME, USA),^14^ and stationed either within the different herds or at the “Les Pyrénéennes” event.

### Description and selection of the azotemic cattle group

To assess the efficacy of SDMA in diagnosing cattle with renal failure secondary to primary kidney diseases, cattle with azotemia, which were referred to the Clinic for Cattle of the National Veterinary School of Toulouse (France), were selected from December 2023 to January 2025. All selected animals underwent assessment of kidney function by measurement of plasma urea and creatinine concentrations, as well as a complete urinalysis (dipstick analysis, USG, urinary cytology, and UPC measurement) at admission to the Clinic for Cattle. To confirm renal involvement, renal ultrasonography was performed, and in the event of death, a necropsy with histologic examination of the kidneys was carried out. The SDMA measurement was performed at a later stage using specimens stored at −20°C or −80°C for less than 1 year before analysis. All analyses except SDMA were performed at the LCBM.

The inclusion criteria were: (1) plasma creatinine and urea concentrations above the upper limit of the laboratory reference interval (ie, above 225 μmol/L for creatinine and above 8.8 mmol/L) for urea, and (2) a final diagnosis of renal disease based on ultrasonography, urinalysis and necropsy with histology in the event of death.

### Blood and urine specimen collection and analysis

For both groups, blood was collected from the coccygeal vein using an 18-gauge needle (Vacutainer and Vacutest; BD Medical) into lithium heparin (4 mL) and EDTA (4 mL) tubes (BD Vacutainer, BD, Plymouth, UK). Throughout the study, specimens were collected and handled by the same operator (C.D.C.) following an identical procedure to minimize preanalytical variability. Specimens underwent centrifugation immediately after collection at 500 × *g* for 15 min (GustoMini centrifuge; Fisherbrand, Heathrow Scientific, IL, USA). Plasma was harvested after centrifugation. Plasma concentrations of urea, creatinine, and SDMA were measured, and the CBC was performed, immediately using the Catalyst One analyzer and the ProCyte Dx in the mobile clinical pathology laboratory. A complete biochemistry panel (urea, creatinine, total protein, albumin, total calcium, inorganic phosphate, magnesium, creatine kinase, AST, and GGT) was performed using a VITROS XT 3400 Chemistry System & Analyzer (QuidelOrtho, San Diego, CA, USA) at the LCBM. All analyses were performed according to the manufacturers’ recommendations and the laboratory’s quality control procedures. All blood analyses were performed within 2 h after sampling, except for linearity and short- and long-term imprecision assessments, for which specimens were stored at −20°C for less than 1 week, and for the complete biochemistry panel, which was performed immediately upon arrival at the LCBM using plasma stored at 4°C for less than 8 h.

Urine specimens were collected from cows by catheterization after careful washing, using a 20 mL syringe (Injekt, B Braun, Melsungen, Germany) and a sterilized catheter, by the same investigator (C.D.C.). For bulls, urine specimens were collected by spontaneous urination. Urine specimens were processed and analyzed within 2 h of sampling, as previously described,^5^ for the following variables: multitest dipstick (Siemens Multistix 10SG, Siemens Healthcare Diagnostics Manufacturing Ltd, Ireland), USG (Master-Sur refractometer, Atago, Tokyo, Japan), UPC, and microscopic examination of the sediment.

Urinary protein and creatinine concentrations were measured on urine samples stored at −20°C for less than a week at the Laboratoire Central de Biologie Médicale using the pyrogallol red (U/CSF Protein, Thermofisher Scientific, Waltham, MA, USA) and enzymatic (Creatinine Enzymatic, Thermofisher Scientific, Waltham, MA, USA) methods, respectively, using an automated analyzer and the manufacturer’s reagents (Indiko, Thermofisher Scientific, Waltham, MA, USA).^5^

### Partial validation of plasma SDMA measurement in cattle

Following ASVCP recommendations,^15^ short-term imprecision (repeatability) was evaluated by 10 consecutive measurements of SDMA in 2 plasma pools with low and high SDMA concentrations. The long-term imprecision (reproducibility) was determined with frozen aliquots from a single plasma pool with a low SDMA concentration. Duplicate measurements were performed in the morning and afternoon for 5 consecutive days.

Linearity was evaluated by analyzing 2 repeats of a 5-point serial dilution (0%, 25%, 50%, 75%, and 100%) prepared from a specimen with high SDMA concentration. The stability of SDMA was assessed using a protocol adapted from a previously published study on SDMA stability in dogs and cats.^16^ The concentration of SDMA was measured in a set of 4 aliquots that had been kept for 1 year at −20°C and −80°C. Plasma aliquots had been immediately analyzed when sampled, then frozen at −20°C or −80°C. One year later, the frozen aliquots were slowly brought to room temperature and carefully homogenized. The SDMA concentration was measured in duplicate and the mean was compared to the initial concentration of SDMA. Mean values of each duplicate measurement were used to estimate the actual concentration of each specimen.

### Calculations and statistics

Linearity was first evaluated visually, then by regression analysis. The comparison of stability results was based on Wilcoxon’s test.

Imprecision was estimated by mean, SD, and the coefficient of variation (CV = SD/mean × 100).

Reference intervals were determined according to ASVCP recommendations^17^ and according to Clinical and Laboratory Standards Institute Evaluation Protocol 28 (CLSI EP 28).^18^ Briefly, distributions were observed to visually detect possible outliers, which then were evaluated using Tukey’s test. Possible effects of covariables (sex, type of production, phase of lactation, and feed) were tested using multifactorial analysis. Because there was no significant effect of the covariables and the number of animals was > 120, the reference intervals and their 90% CIs were estimated by a nonparametric method.^19^

The diagnostic efficacy of SDMA was based on the calculation of sensitivity and specificity (ie, the rates of true positives and true negatives), using the upper limit of the reference interval as the cut-off. The receiver operating characteristic (ROC) curve of SDMA efficacy was obtained by plotting sensitivity vs (1 − specificity) according to increasing cut-offs, and the area under the curve (AUC) was determined.

Descriptive statistics and reference intervals with their 90% CI were calculated using Reference Value Advisor freeware.^20^ Other calculations used Systat (Systat Software, San Jose, CA), Analyse-It (v. 2.30, Analyse-It Software, Ltd., UK), and Excel (Microsoft, Redmond, WA); significance was set at *P* < .05.

## Results

### Analytical characteristics of SDMA measurement in bovine plasma

#### Linearity

**Figure 1:**
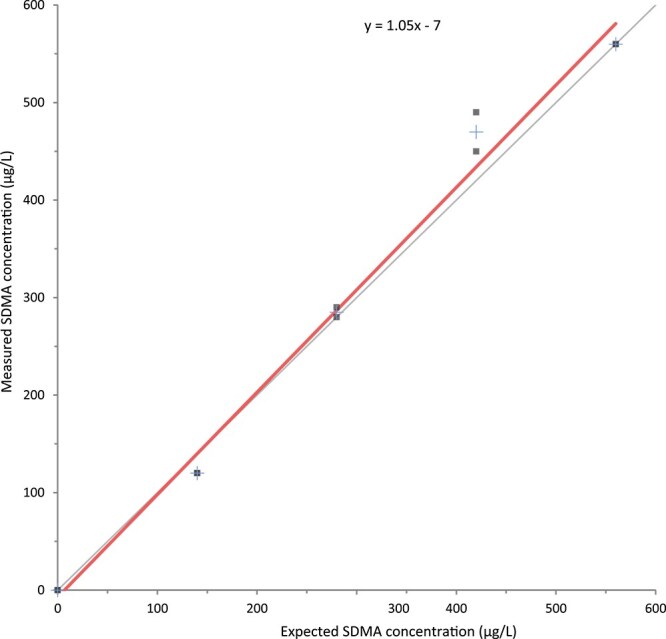
Linearity of SDMA concentration under dilution. Squares represent results from duplicate analyses, and crosses represent the mean of duplicates. Red line represents linear fit, and gray line represents the identity line (x = y). x: Expected SDMA concentration (μg/L); y: Measured SDMA concentration (μg/L).

Linearity was visually good (maximum difference from linear fit ≤ 12%), and linear fitting (*R*^2^) was > 0.99 (Figure 1).

#### Imprecision

The coefficient of variation (CV) of short-term imprecision (repeatability) from 10 consecutive replicates was 11.8% and 9.2% at mean concentrations of 73 and 349 μg/L, respectively. The CV of long-term imprecision (reproducibility) from daily replicates morning and afternoon for 5 consecutive days was 7.6%, with a mean concentration of 78 μg/L.

#### Stability

Plasma SDMA concentration was not different from T0 in specimens stored for one year at −20°C and −80°C (Wilcoxon test, *P* = .5; Figure S1).

### Plasma SDMA reference interval

The reference sample group (*n* = 199) included 50 dairy cows (40 Montbeliarde, 10 Prim’ Holstein) and 149 beef cattle (53 males and 96 females; 43 Aubrac, 5 Bazadaise, 23 Blonde d’Aquitaine, 23 Charolaise, 17 Gasconne, and 38 Limousine), that were fed GS (52), CS (45), G (90), and H (12) or some combination of these.

**Figure 2:**
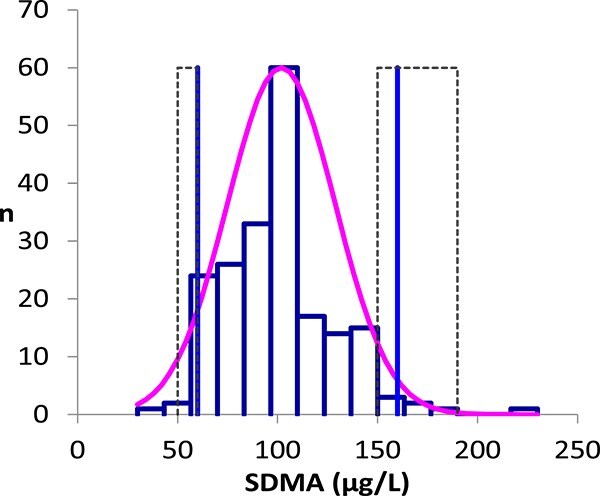
Distribution of SDMA concentration in 199 adult cattle (curve is the fitted distribution; vertical lines are the limits of the reference interval with 90% CIs as dotted lines).

No significant effect of sex, type of production, or diet was found on SDMA concentration (analysis of variance; *P* > .17). Visual inspection of SDMA distribution (Figure 2) showed 2 possible outliers, which were not confirmed by Tukey’s test and were retained. The distributions of native and Box–Cox transformed values were significantly different from a Gaussian distribution (Anderson–Darling tests, *P* < .001). Median and minimum–maximum interval were 100 and 30-230 μg/L, respectively. The upper and lower limits of the reference interval determined using the nonparametric method were 60 (90% CI, 50-60) and 160 (90% CI, 150-190) μg/L, respectively.

### Diagnostic efficacy of SDMA

Among the 27 azotemic cattle, 23 cows were diagnosed with bilateral pyelonephritis, 1 cow and 1 bull with bilateral tubular necrosis, and 2 cows with bilateral glomerulonephritis. Tubular necrosis and glomerulonephritis cases were diagnosed by histopathology. Pyelonephritis cases were diagnosed either by renal ultrasonography combined with urinalysis, by histopathology, or by both methods. Overall, diagnoses were established by histopathology in 55.6% of cases, and by renal ultrasonography combined with urinalysis in the remaining 44.4% (Table 1).

**Table 1:** Causes of renal disease identified in the 27 azotemic cattle and diagnostic methods employed, including the number of affected animals, ranges of plasma urea, creatinine and symmetric dimethylarginine (SDMA) concentrations, renal ultrasonography, urinalysis with urine specific gravity (USG), and histopathology findings at necropsy.

**Diagnosis**	**Number**	**Urea (mmol/L)**	**Creatinine (μmol/L)**	**SDMA (μg/L)**	**Renal ultrasonography**	**Urinalysis**		**Histopathology—necropsy**
					**Number**	**Number**	**USG**	**Number**
**Pyelonephritis**	23	14.8-80.3	476-3102	140-1000	23	23	1.002-1.020	11
**Tubular necrosis**	2	39.8-95.2	1130-1773	250-580	2	2	1.010-1.012	2
**Glomerulonephritis**	2	40.8-68.0	468-993	340-370	2	2	1.010-1.020	2

In azotemic cattle, plasma SDMA concentrations ranged from 140 to 1000 μg/L. Although most results exceeded the calculated reference interval (60-160 μg/L), 3 animals had SDMA concentrations within the reference interval, 1 was equal to the upper reference limit, and 1 fell within the CI of the upper reference limit.

**Figure 3:**
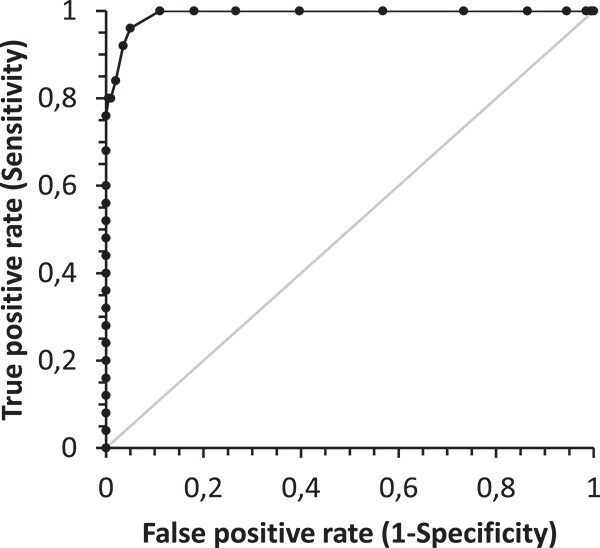
ROC curve of plasma SDMA concentration for the diagnosis of renal diseases in cattle.

The diagnostic efficacy of SDMA for kidney diseases in cattle was excellent. The AUC of the sensitivity vs (1 − specificity) curve (Figure 3) was 0.993 (0.986-1.000). The intercept of the Northwest-Southeast diagonal indicates 140 μg/L as the concentration of plasma SDMA having the highest rate of true positives and true negatives (0.96 and 0.95, respectively). Using the upper limit of the reference interval (160 μg/L), as a cut-off, sensitivity and specificity were 0.85 (90% CI, 0.66-0.96) and 0.98 (90% CI, 0.95-0.99). With this same cut-off, the positive predictive value of a positive result (ie, SDMA > 160 μg/L) reached 0.95 for a pretest probability equal to 0.31, and was equal to 0.98 at pretest probability of 0.50 (Figure S2).

## Discussion

We have evaluated the main analytical characteristics of SDMA measurement in bovine plasma using the Catalyst One analyzer, to establish a de novo reference interval for plasma SDMA concentrations in a large cattle population (including bulls, dairy cows, and beef cows) and to confirm its diagnostic effectiveness for detecting renal disease in cattle confirmed by histopathological examination, urinalysis combined with ultrasonographic findings, or both.

Studies investigating the imprecision and stability of SDMA concentrations using the Catalyst One analyzer are limited.^21–23^ Most available data in cats and dogs focus on results obtained using liquid chromatography-mass spectrometry (LC–MS)^22^ or the enzyme multiplied immunoassay technique (EMIT) used by the IDEXX reference laboratory.^16^ In our study, both short-term and long-term imprecision values were close to the analytical performance goals for SDMA using the Catalyst One analyzer (Imprecision < 10%).^24^ Imprecision reported in cats and dogs using the Catalyst analyzer^16^^,^^23^^,^^25^ range from 4.8% to 11.61%, results similar to those obtained in our study.

The long-term stability of SDMA measured using the Catalyst One analyzer has not yet been investigated.^16^ Available studies using this analyzer have only assessed short-term storage in dogs and cats, demonstrating stability for up to 24 h at room temperature and for up to 7 days at 4°C.^16^ In contrast, long-term stability studies (up to 2 years at −24°C and −80°C) have only been performed using the EMIT method, confirming SDMA stability under these conditions.^16^ In our study, SDMA was stable in bovine plasma stored for up to 1 year under similar conditions and measured using the Catalyst One analyzer.

Two previous studies have reported plasma SDMA concentrations in cattle.^26^^,^^27^ However, the aims and study designs of these studies differed from those of our study, which makes direct comparisons difficult. In these previous studies, healthy cows were included primarily as control animals rather than for the purpose of establishing reference intervals. Consequently, reported SDMA results in healthy cows should not be interpreted as formal reference intervals. Nevertheless, when comparing the reported SDMA results, both studies reported narrower distributions and generally lower concentrations than those observed in our study. These differences may partly reflect differences in study design and population characteristics.^28^^,^^29^ Despite these differences, those studies provide useful information for SDMA interpretation in dairy cattle. In particular, breed differences should be considered, because Brown Swiss cows have been reported to have higher serum SDMA concentrations than Holstein cows, reflecting differences in renal function among breeds. In addition, SDMA concentrations do not appear to differ between healthy cows and cows with mastitis, suggesting that this condition is unlikely to influence SDMA interpretation. When comparing the reference interval established in cattle with those reported in other species following international guidelines, only a limited number of studies are available. In our study, no significant influence of extra-renal factors such as sex, production type, breed, or dietary supply on SDMA concentrations was observed. This finding is consistent with reports in dogs^4^ and horses,^11^^,^^30^ but contrasts with the results of 1 study in cattle,^27^ which observed higher SDMA concentrations in 31 Brown Swiss cows compared with 46 Holstein cows. This discrepancy may, in part, be related to the fact that urinalysis and clinical examinations were not performed in that study to exclude cows with subclinical disease. More importantly, no Brown Swiss cows were included in our study. Because Brown Swiss cows have higher serum urea and creatinine concentrations than Holstein cows kept under identical dietary, housing, and health conditions, it is plausible that their higher SDMA concentrations reflect breed-specific differences in renal function, potentially linked to a lower GFR.^27^

The diagnostic performance of SDMA for detecting kidney disease in cattle was excellent at a threshold of 160 μg/L. This value is similar to thresholds reported in other species, including horses (190 μg/L),^31^ cats (180 μg/L),^21^ and dogs (160-180 μg/L),^32^^,^^33^ as determined using enzyme immunoassays in IDEXX reference laboratories for the detection of abnormal GFR or acute kidney injury. However, the sensitivity and specificity of SDMA are not consistently high and can vary across species and studies. According to one study,^12^ SDMA is not a sensitive biomarker for urinary obstruction in sheep and goats and shows no correlation with serum creatinine concentration. Furthermore, in some types of cancer (lymphomas, carcinomas, and sarcomas), SDMA can be increased, potentially because of overproduction by tumor tissue, as observed in dogs and cats.^34^^,^^35^ Therefore, additional studies are required to determine whether SDMA provides greater sensitivity than creatinine for detecting renal dysfunction and to clarify the correlation between SDMA concentration and GFR in cattle.

A limitation of our study is the incomplete validation process. In particular, long-term imprecision was assessed using only a single plasma pool with an SDMA concentration within the reference interval. Linearity was evaluated by analyzing 2 replicates of a 5-point serial dilution, rather than the recommended 3 replicates. Although a partial analytical validation supported the measurement of SDMA in bovine plasma using the Catalyst One analyzer, additional comparison with a reference method such as LC–MS would be valuable in future studies. Another limitation of our study is its retrospective design, because the analyses were performed on frozen samples. Although stability testing indicated that SDMA remains stable, a prospective study using fresh plasma would have been preferable. Furthermore, the diagnostic efficacy assessment was based only on azotemic cattle with an etiologic diagnosis vs healthy cattle. The specificity of a diagnostic test can be estimated using either a population of healthy animals or a population of animals affected by diseases other than the condition targeted by the test. In the latter case, specificity and positive predictive value often are decreased because prerenal azotemia, which is common in sick cattle, may confound test results even in the absence of kidney disease.^36^ To avoid this confounding effect, we elected to first evaluate diagnostic performance in a population of healthy animals only.

## Conclusion

Symmetric dimethylarginine concentrations showed acceptable analytical performance and stability. No significant effect of sex, production type, or diet was observed on SDMA concentration in healthy cattle. Although these are preliminary results, the upper reference limit of 160 μg/L demonstrated high sensitivity and specificity for detecting azotemia, confirming SDMA as a potentially useful renal biomarker in cattle. Additional studies are needed to explore its correlation with GFR and sensitivity in early kidney disease.

## Supplementary Material

Supplementary_figure_1_aalag108

Supplementary_figure_2_aalag108

## Off-label antimicrobial declaration

The authors declare no off-label use of antimicrobials.

## Institutional animal care and use committee or other approval declaration

The protocol was approved by the Science et Santé Animale ethics committee (SSA-2025-011_V3). A consent form was signed by all breeders.

## Abbreviations

ASVCP: American Society of Veterinary Clinical Pathology
CV: Coefficient of variation
CS: Corn silage
GS: Grass silage
GFR: Glomerular filtration rate
H: Hay
SDMA: Symmetric dimethylarginine
UPC: Urine protein-to-creatinine ratio
USG: Urine specific gravity

## References

[1] Rosenbaum A, Guard CL, Njaa BL, et al. Slaughterhouse survey of pyelonephritis in dairy cows. Vet Rec. 2005;157:652-655. 10.1136/vr.157.21.652 16299366

[2] Válková L, Voslářová E, Becskei Z, Večerek V. Comparison of the incidence of kidney damage in cattle, pigs, sheep and goats detected at slaughterhouses as an indicator of animal health. Acta Vet Brno. 2023;92:321-328. 10.2754/avb202392030321

[3] Defontis M, Bauer N, Failing K, Moritz A. Automated and visual analysis of commercial urinary dipsticks in dogs, cats and cattle. Res Vet Sci. 2013;94:440-445. 10.1016/j.rvsc.2013.01.002 23360686

[4] Hokamp JA, Nabity MB. Renal biomarkers in domestic species. Vet Clin Pathol. 2016;45:28-56. 10.1111/vcp.12333 26918420

[5] Herman N, Bourgès-Abella N, Braun JP, Ancel C, Schelcher F, Trumel C. Urinalysis and determination of the urine protein-to-creatinine ratio reference interval in healthy cows. J Vet Intern Med. 2019;33:999-1008. 10.1111/jvim.15452 30768734 PMC6430871

[6] van GG, Olsen E, Siwinska N. Biomarkers of kidney disease in horses: a review of the current literature. Animals. 2022;12:2678. 10.3390/ani12192678 36230418 PMC9559299

[7] Pérez-López L, Boronat M, Melián C, Santana A, Brito-Casillas Y, Wägner AM. Short-term evaluation of renal markers in overweight adult cats. Vet Med Sci. 2023;9:572-578. 10.1002/vms3.1021 36639961 PMC10029907

[8] Melendez P, Donovan A, Hernandez J, et al. Milk, plasma, and blood urea nitrogen concentrations, dietary protein, and fertility in dairy cattle. J Am Vet Med Assoc. 2003;223:628-634. 10.2460/javma.2003.223.628 12959380

[9] Bindi F, Nocera I, Meucci V, et al. Symmetric and asymmetric dimethylarginines in healthy and colic horses. Res Vet Sci. 2025;188:105615. 10.1016/j.rvsc.2025.105615 40117953

[10] Lo HC, Winter JC, Merle R, Gehlen H. Symmetric dimethylarginine and renal function analysis in horses with dehydration. Equine Vet J. 2022;54:670-678. 10.1111/evj.13484 34110650

[11] Schott HC, Gallant LR, Coyne M, et al. Symmetric dimethylarginine and creatinine concentrations in serum of healthy draft horses. J Vet Intern Med. 2021;35:1147-1154. 10.1111/jvim.16042 33543506 PMC7995414

[12] Camacho BE, Mitman SL, Foster DM, Halleran J. Validation of a reference interval for symmetric dimethylarginine in healthy goats and its comparison to values in goats with obstructive urolithiasis. J Vet Intern Med. 2024;38:2807-2813. 10.1111/jvim.17162 39152724 PMC11423465

[13] Kiene F, Stöter M, Ganter M. O-057 symmetric dimethylarginine as an early serum parameter to detect kidney injury in sheep and goats. Anim Sci Proc. 2023;14:97. 10.1016/j.anscip.2023.01.133

[14] Chenal T, Lambert M, Prieux A, et al. Validation of a mobile clinical pathology laboratory for canine hematology and biochemistry. BMC Vet Res. 2025;21:283. 10.1186/s12917-025-04601-6 40264110 PMC12013186

[15] Arnold JE, Camus MS, Freeman KP, et al. ASVCP guidelines: principles of quality assurance and standards for veterinary clinical pathology (version 3.0). Vet Clin Pathol. 2019;48:542-618. 10.1111/vcp.12810 31889337

[16] Brans M, Marynissen S, Mortier F, Duchateau L, Daminet S, Paepe D. Effect of storage temperature and time on measurement of serum symmetric dimethylarginine concentration using point-of-care and commercial laboratory analyzers in cats and dogs. J Vet Intern Med. 2023;37:1794-1805. 10.1111/jvim.16811 PMC10472997 37565515

[17] Friedrichs KR, Harr KE, Freeman KP, et al. ASVCP reference interval guidelines: determination of de novo reference intervals in veterinary species and other related topics. Vet Clin Pathol. 2012;41:441-453. 10.1111/vcp.12006 23240820

[18] Horowitz GL . Defining, Establishing, and Verifying Reference Intervals in the Clinical Laboratory: Approved Guideline. 3rd ed. Clinical and Laboratory Standards Institute; 2008.

[19] Ozarda Y . Reference intervals: current status, recent developments and future considerations. Biochem Med. 2016;26:5-16. 10.11613/BM.2016.001 PMC4783089 26981015

[20] Geffré A, Concordet D, Braun JP, Trumel C. Reference value advisor: a new freeware set of macroinstructions to calculate reference intervals with Microsoft excel. Vet Clin Pathol. 2011;40:107-112. 10.1111/j.1939-165X.2011.00287.x 21366659

[21] Brans M, Daminet S, Mortier F, Duchateau L, Lefebvre HP, Paepe D. Plasma symmetric dimethylarginine and creatinine concentrations and glomerular filtration rate in cats with normal and decreased renal function. J Vet Intern Med. 2021;35:303-311. 10.1111/jvim.15975 33274800 PMC7848354

[22] Prieto JM, Carney PC, Miller ML, et al. Biologic variation of symmetric dimethylarginine and creatinine in clinically healthy cats. Vet Clin Pathol. 2020;49:401-406. 10.1111/vcp.12884 32716076

[23] Baral RM, Freeman KP, Flatland B. Comparison of serum and plasma SDMA measured with point-of-care and reference laboratory analysers: implications for interpretation of SDMA in cats. J Feline Med Surg. 2021;23:906-920. 10.1177/1098612X20983260 33544013 PMC11197123

[24] Baral RM, Freeman KP, Flatland B. Analytical quality performance goals for symmetric dimethylarginine in cats. Vet Clin Pathol. 2021;50:57-61. 10.1111/vcp.12951 33524207

[25] Halman C, Courtman N, Stone B. Comparison of 2 point-of-care analyzers and the Eurolyser assay with an IDEXX reference laboratory method for measurement of symmetric dimethylarginine in dogs. Am J Vet R. 2025;86:1–12. 10.2460/ajvr.24.07.0204 39892400

[26] Bronzo V, Sala G, Ciabattini I, et al. Endogenous symmetric dimethylarginine (SDMA) and asymmetrical dimethylarginine (ADMA) levels in healthy cows and cows with subclinical and clinical mastitis—a comparative study. Animals (Basel). 2025;15:527. 10.3390/ani15040527 PMC11851524 40003009

[27] Kessler EC, Bruckmaier RM, Gross JJ. Kidney function, but not nitrogen excretion differs between Brown Swiss and Holstein dairy cows. J Dairy Sci. 2024;107:8736-8745. 10.3168/jds.2024-24997 38908706

[28] Cozzi G, Ravarotto L, Gottardo F, et al. Short communication: reference values for blood parameters in Holstein dairy cows: effects of parity, stage of lactation, and season of production. J Dairy Sci. 2011;94:3895-3901. 10.3168/jds.2010-3687 21787926

[29] Guyot H, Legroux D, Eppe J, Bureau F, Cannon L, Ramery E. Hematologic and serum biochemical characteristics of Belgian blue cattle. Vet Sci. 2024;11:222. 10.3390/vetsci11050222 38787194 PMC11125627

[30] Siwinska N, Zak A, Slowikowska M, Niedzwiedz A, Paslawska U. Serum symmetric dimethylarginine concentration in healthy horses and horses with acute kidney injury. BMC Vet Res. 2020;16:396. 10.1186/s12917-020-02621-y 33081772 PMC7576750

[31] Siwinska N, Zak A, Paslawska U. Detecting acute kidney injury in horses by measuring the concentration of symmetric dimethylarginine in serum. Acta Vet Scand. 2021;63:3. 10.1186/s13028-021-00568-0 PMC7809759 33446216

[32] McKenna M, Pelligand L, Elliott J, Cotter D, Jepson R. Relationship between serum iohexol clearance, serum SDMA concentration, and serum creatinine concentration in non-azotemic dogs. J Vet Intern Med. 2020;34:186-194. 10.1111/jvim.15659 31725186 PMC6979102

[33] Pelander L, Häggström J, Larsson A, et al. Comparison of the diagnostic value of symmetric dimethylarginine, cystatin C, and creatinine for detection of decreased glomerular filtration rate in dogs. J Vet Intern Med. 2019;33:630-639. 10.1111/jvim.15445 30791142 PMC6430914

[34] Rixon A, Meyer E, Daminet S, Goddard A, Kongtasai T, Pazzi P. Influence of carcinoma and sarcoma on neutrophil gelatinase-associated lipocalin and symmetric dimethylarginine concentrations in dogs. J Vet Intern Med. 2025;39:e70015. 10.1111/jvim.70015 40042235 PMC11881161

[35] Coyne MJ, Drake C, McCrann DJ, Kincaid D. The association between symmetric dimethylarginine concentrations and various neoplasms in dogs and cats. Vet Comp Oncol. 2022;20:846-853. 10.1111/vco.12845 35718995

[36] Braun JP, Concordet D, Lyazrhi M, Lefebvre HP, Toutain PL. Overestimation of the predictive value of positives by the usual calculations of the specificity of diagnostic tests. Vet Res Commun. 2000;24:17-24. 10.1023/A:1006369120260 10703750

