# Supplementary material for: Analytical validation, reference interval determination, and diagnostic performance of plasma symmetric dimethylarginine in cattle
Source: J Vet Intern Med. 2026 Jun 9;40(3):aalag108. doi: 10.1093/jvimsj/aalag108 (PMC13249082; doi:10.1093/jvimsj/aalag108)
Supplement: Supplementary_figure_1_aalag108 [file supplementary_figure_1_aalag108.pdf]

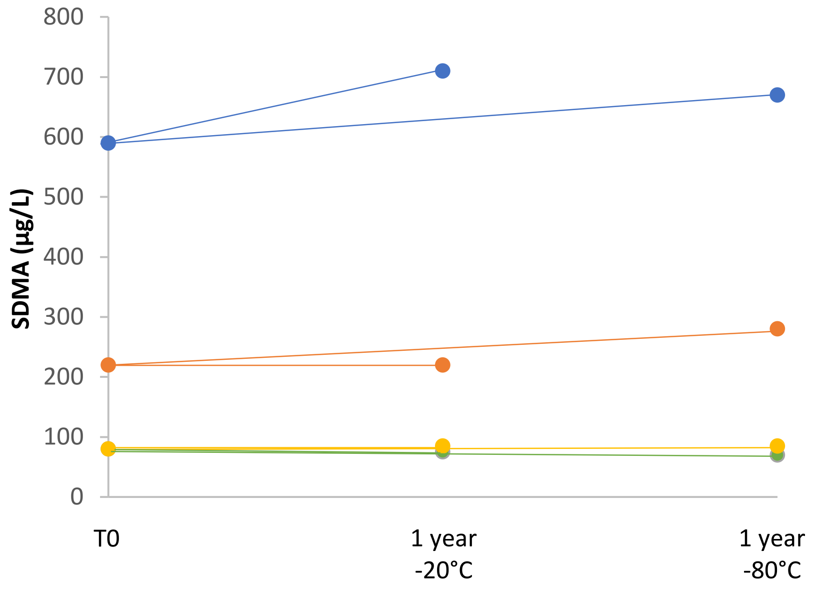

Supplementary figure 1: Stability of bovine plasma SDMA concentration after 1-year storage at -20 and -80°C
