# Supplementary material for: Analytical validation, reference interval determination, and diagnostic performance of plasma symmetric dimethylarginine in cattle
Source: J Vet Intern Med. 2026 Jun 9;40(3):aalag108. doi: 10.1093/jvimsj/aalag108 (PMC13249082; doi:10.1093/jvimsj/aalag108)
Supplement: Supplementary_figure_2_aalag108 [file supplementary_figure_2_aalag108.pdf]

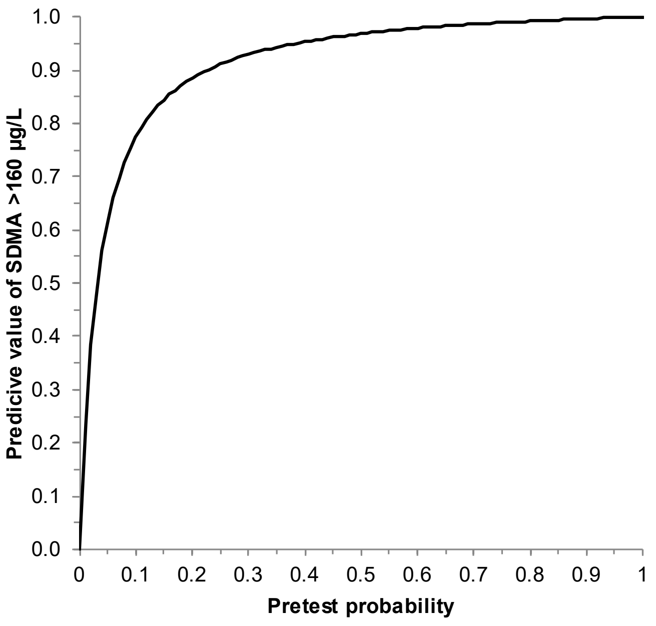

Supplementary figure 2 : Variation of the positive predictive value of SDMA > 160 µg/L for the diagnosis of bovine kidney diseases according to the value of the pre-test probability.
